# The impact of parturition induction with cloprostenol on immunological parameters in the sow colostrum

**DOI:** 10.1186/s40813-020-00174-y

**Published:** 2020-12-11

**Authors:** Karolina Hlavová, Hana Kudláčková, Martin Faldyna

**Affiliations:** Veterinary Research Institute, Hudcova 296/70, 621 00 Brno, Czech Republic

**Keywords:** Sow, Colostrum, Lymphocytes, Cytokines, Complement, Lysozyme

## Abstract

**Background:**

Farrowing induction with prostaglandin F2 analogue cloprostenol is commonly used on commercial farms to manage the timing of farrowing. When labour induction is applied, the questions arise about possible side effects of such a hormonal intervention on physiological processes connected with labour and lactation, including colostral immunity.

**Results:**

In this study, immune cells composition, lysozyme concentration, complement bacteriolytic activity and proinflamatory (GM-CSF2, IL-1β, IL-6, a TNFα) and anti-inflammatory (IL-4, IL-10, TGFβ1 a TGFβ2) cytokines were measured in colostrum samples from sows farrowing naturally (NP) and from sows with farrowing induced using cloprostenol administration on day 113 of gestation (IP). A significantly higher proportion of lymphocytes was found in colostrum of induced sows compared to colostrum of non-induced sows. No significant differences between NP and IP were found in complement activity, in the proportions of granulocytes, macrophages and lymphocyte subpopulations. Lower lysozyme concentration and higher IL-1β, IL-6, TGFβ1 and TNFα concentrations were found in IP sow colostrum compared to colostrum from NP sows.

**Conclusions:**

An increased proportion of colostral lymphocytes can positively influence the cellular immunity transmission from sow to her offspring. On the other hand, a lower lysozyme concentration can adversely affect newborn’s intestinal immunity, as well as changes in cytokine concentrations can have an adverse effect on newborn piglet intestinal epithelium development and its defence function.

## Background

Parturition induction using prostaglandin F2α (PGF2α) or its synthetic analogue cloprostenol is a common practice in pig industry [1, 2]. This animal husbandry intervention has many positive effects on breeding efficiency. Parturition induction on its own decreases variability in gestation length, facilitating the timing of birth. This makes it easier to look after the sow and newborn piglets during the birth and control the sufficient intake of colostrum by all piglets. More careful birth monitoring leads to lower number of stillbirths and neonatal mortality [3] and increases total colostrum intake by whole litter [4]. However, in spite of the above mentioned positive effects, questions are arising, whether parturition induction could adversely affect various physiological parameters, e.g. immunoglobulins content, presence of immune cells etc.

For a newborn piglet, colostrum is a vital source of immunologically active substances, which play the main role in piglet’s protection against potential infection. Colostrum components also help a newborn piglet to adapt to living conditions after leaving the sterile environment in the uterus. The epitheliochorial placenta of the pig does not allow transmission of antibodies from mother’s to piglet’s circulation in utero. Therefore, careful attention is given to colostral antibodies. A shortage of colostrum antibodies is life threatening for the newborn piglet [5]. Concerning immunoglobulin G (IgG) concentration in colostrum, the impact of parturition induction was not found in colostrum of primiparous sows [6]. However, lower levels of IgG were found in colostrum of sows with cloprostenol induced parturition as well as in their piglets’ sera, compared to sows with non-induced parturition [7].

Besides antibodies, colostrum contains a variety of immune cells and immunologically active compounds which have a direct antimicrobial effect (e.g. complement, lysozyme) or induce and modulate the immune response (e.g. cytokines, growth factors) [8].

Immune cells in porcine colostrum are mainly phagocytes and lymphocytes, out of which 70–90% are represented by T cells [9, 10]. Colostral T cells show more effector or memory phenotypic features compared to T cells found in blood [11], are able to migrate into piglet tissues and respond to specific antigen stimulation [12]. Together with cells, colostrum contains many humoral compounds. The main role in humoral immunity is played by complement and lysozyme. The complement system comprises more than sixty components and activation fragments. It is a part of the innate immune system, it defends the body mainly against gram-negative bacteria, removes immune complexes, contributes to organ regeneration and engages T and B cells of the adaptive immune response [13]. Many studies have shown the presence of its haemolytic or bactericidal activity in bovine colostrum [14–17]. However, to the author’s knowledge, there is no information about the complement content and activity in porcine colostrum. Lysozyme is 1,4,-β-N-acetylmuramidase, an enzyme, which cleaves the glycosidic bonds in bacterial cell wall peptidoglycans [18, 19]. It increases the gut resistance and protects against a wide spectrum of gram-positive and gram-negative bacteria (e.g. enterotoxigenic E.coli). The addition of lysozyme to feed leads to increased weight gains with similar results as global subtherapeutic antibiotic use, which is widely being reduced [20, 21].

Passively acquired cytokines modulate the secretion of other cytokines by splenocytes and affect the differentiation of the newborn’s immune cells [22]. Porcine colostrum contains a substantial amount of transforming growth factor β (TGF-β) [23]^.^ The presence and the role of other cytokines in porcine colostrum is poorly documented, regardless of their presumable impact on piglet development and immunity.

The aim of this study was to describe the impact of induction of parturition on selected immunological parameters (immune cells, complement, lysozyme, cytokines) in porcine colostrum with regard to their important impact on the newborn piglet immune system development. The proportion of immune cells (granulocytes, macrophages and lymphocytes with their subpopulations), complement bacteriolytic activity, concentration of lysozyme and cytokines – proinflammatory: granulocyte-macrophage colony-stimulating factor 2 (GM-CSF 2), interleukin 1β (IL-1β), interleukin 6 (IL-6) and tumour necrosis factor α (TNFα) and also antiinflammatory: interleukin 4 (IL-4), interleukin10 (IL-10), TGFβ 1 and TGFβ 2 – was determined in colostrum of sows in which parturition was induced by cloprostenol and of those which delivered spontaneously.

## Results

### Total leukocyte counts

The analysis of cells based on propidium iodide exclusion indicated mean cell viability of 95.01% ± 4.89 of isolated cells. The proportion of lymphocytes from all CD45^+^ leucocytes was significantly (*P* = 0.0064) higher in colostrum of induced sows (32.0% ±16.3) compared to colostrum of non-induced sows (13.8% ±10.5). It was compensated by lower percentages of granulocytes (51.3% ±15.8 in induced sows and 54.8% ±14.2 in non-induced sows) and macrophages (12.0% ±5.0 in induced sows and 21.3% ±17.8 in non-induced sows).

### Lymphocyte subpopulations

The most abundant lymphocyte subpopulation was Tc cells (induced sows: 33.3% ± 8.2; non-induced sows: 35.8% ± 11.3) followed by γδ cells (induced sows: 30.8% ± 12.2; non-induced sows: 22.7% ± 7.6), DP cells (induced sows: 21.3% ± 9.3; non-induced sows: 24.8% ± 8.0) and NK cells (induced sows: 11.2% ± 3.8; non-induced sows: 10.4% ± 6.4), with a relatively low contribution of Th cells (induced sows: 2.1% ± 2.5; non-induced sows: 2.5% ± 2.4). No significant differences were found in the proportions of lymphocyte subpopulations comparing colostrum from induced and non-induced sows.

### Lysozyme concentration

In samples from sows with induced parturition, the concentration of lysozyme was significantly (*p* = 0.0032) lower (0.254 ± 0.082 mg/ml) compared to concentrations found in samples from sows with non-induced parturition (0.424 ± 0.167 mg/ml).

### Complement activity

As a measure of the complement activity in colostrum supernatant, a reciprocal of the time of killing 50% of the bacterial population (h^− 1^) was determined. In samples from sows with induced parturition, the complement activity was 1.158 ± 0.223 h^− 1^ and from sows with non-induced parturition 1.164 ± 0.211 h^− 1^. There was not found any significant difference between samples from induced and non-induced sows.

### Cytokines concentrations

The levels of GM-CSF2, Il-1β, Il-4, Il-6, Il-10, TGFβ1, TGFβ2 and TNFα were measured in supernatants from all colostrum samples. High variances among different samples were found in concentrations of all cytokines. Increased levels of all measured cytokines were noted in colostrum samples from sows with induced parturition and significantly higher levels of Il-1β (*p* = 0.0035), Il-6 (*p* = 0.0155), TGFβ1 (*p* = 0,0366) and TNFα (*p* = 0.0216) were found. Data for all cytokines are summarised in Table 1.

**Table 1:** Cytokine concentrations (pg/ml) in colostral supernatant from sows with induced parturition (IP, *n* = 19) and with spontaneous non-induced parturition (NP, *n* = 18). The values are expressed as mean +/−SD. Cytokines showing significant differences (*p* ≤ 0.05) between IP and NP are marked with asterisks

	GM-CSF2	IL-1β *	IL-4	IL-6 *	IL-10	TGFβ1 *	TGFβ2	TNFα *
**IP (mean)**	116.09	4596.42	874.44	17,117.67	104.42	238.32	75.79	4469.00
**SD**	261.18	2850.11	738.62	9140.57	414.50	393.73	321.55	4987.24
**NP (mean)**	106.47	1936.89	464.00	9679.33	30.51	15.78	9.89	3838.13
**SD**	160.28	2119.70	372.31	9900.21	93.19	65.05	40.77	12,347.85

## Discussion

Many studies confirmed the importance of careful supervision of the sow and newborn piglets during parturition and at an early time after. One of the most important tasks is to arrange an equal access to colostrum for all piglets from the litter [3]. More uniform time of parturition as a consequence of induction enables us to concentrate the majority of farrowings into the working hours of the farm staff. Neonatal assistance with colostrum intake supervision and cross-fostering increase the chances for survival of later born and smaller piglets [24]. In spite of these advantages, concerns are arising among breeders and veterinarians as to whether the farrowing induction by cloprostenol affects colostrum quality and potentially the immunity of the piglets. In this study, we focused on selected immunological compounds in colostrum from sows with cloprostenol-induced parturition and from sows with spontaneous farrowing. The studied parameters were: the proportion of immune cells, complement activity, concentration of lysozyme and cytokines.

The colostrum contains substantial numbers of immune cells which are known to be able to act directly in the mammary gland and in piglet intestinal lumen (neutrophils, macrophages), or can, in the early days of life, migrate into the piglet tissues where they can play a role in adaptive immunity [9, 12, 25]. This study demonstrated a significantly higher proportion of lymphocytes in colostrum of sows with induced parturition, maintaining the same lymphocyte subpopulations distribution, from which we can infer that the migration of cells to the mammary gland caused by induction is not selective. As the PGF2α is able to modulate the expression of chemokines and their receptors [26], the increased proportion of lymphocytes could be explained by possible increased secretion of chemokines which are responsible for the recruitment of cells into the lactating mammary gland [8, 27]. The ELR+ CXC chemokine gene promoter sequences have DNA binding sites for the inducible transcription factors AP-1, C/EBP and NF-kB, which in turn are regulated by multiple stimuli including endocrine factors such as prolactin, progesterone and inflammatory cytokines (IL-1, TNF-a) [28]. And these factors are also modulated by PGF2α [29–31]. We also confirmed the data from our previous study [11] that the majority of colostral leukocytes are cytotoxic T cells. In a mouse model, the majority of colostral cytotoxic T cells (CTLs) exhibited high levels of the gut-homing molecules α4β7 and CCR9, but a reduced expression of the systemic homing marker CD62L. Under the same activation conditions, CD8 T cells transferred through colostrum have a superior capacity to produce potent cytolytic and inflammatory mediators when compared to those generated by the breastfed infant [32], which means that the increased proportion of lymphocytes induced by cloprostenol treatment could potentiate the response.

The complement bacteriolytic activity was determined via bioluminescence assay in this study. It was found that the activity was similar in both studied groups. To the authors***’*** knowledge, there is currently no information about the complement activity detection in porcine colostrum. According to Maye [33] the bactericidal activity in bovine milk is higher than in human milk. Similarly, Sakai [34] detected a lower complement activity in porcine sera compared to human sera. Each of the above mentioned authors used a different method of complement activity determination, so itis incomparable in absolute numbers. However, it can help general comparison among species, and further comparative studies of complement activity would be contributive.

According to the results of this study, it was proven that cloprostenol treatment significantly decreases the concentration of lysozyme in colostrum. The mechanism of lysozyme decrease is possibly via cloprostenol induced progesterone drop [35] which besides other effects affects lysozyme activity [36]. Lysozyme as an antibacterial enzyme plays a very important role in non-specific immunity and is necessary for complement binding to the secreted IgA [9]. In case of a decreased level of lysozyme, the barrier function of the gut mucosa can be reduced.

The cytokine analysis in this study revealed higher concentrations of IL-1β and TNFα in colostrum from sows with parturition induced by cloprostenol, compared to colostrum from sows with non-induced parturition. It is known that PGF2α is able to induce acute inflammation including the increase of above mentioned pro-inflammatory cytokines [31]. In human colostrum, IL-1β stimulates the secretion of TNFα by monocytes and helper T cells, leading to the production of pro-inflammatory cytokines stimulating macrophages and consequently establishing cellular immunity in newborns [37]. TNFα by itself stimulates mucin secretion in gut epithelia [38]. As opposed to pro-inflammatory cytokines, higher levels of IL-6 and TGFβ1 were found in colostrum of sows with IP. Both of them regulate pro-inflammatory cytokine production [39] and stimulate antibody secretion, mainly mucosal IgA, as in milk cells as in the newborn’s intestine [40, 41]. The largest amount of cytokines is absorbed into the offspring circulation before the process designated as gut closure takes place. It is about 1–2 days after birth, at the same time when mammary secretion changes from colostrum to milk [42]. Maternal cytokines transferred to the offspring via colostrum play an essential role as mediators of early response to infection and modulate the immune repertoire of the offspring [22]. It is assumed that TGFβ plays a physiological role in regulation of postnatal adaptation of the newborn’s gastrointestinal tract [23]. Gut mucosa epithelial cells have on their apical surface many receptors which react to cytokines produced by neighbouring cells (e.g. enterocytes, immune cells). In the newborn intestinal mucosa, epithelial cells also respond to cytokines entering the intestinal lumen with colostrum. To give an example, proinflammatory cytokines (IL-1) can potentiate the inflammatory response of gut epithelia [43]. Many studies on cellular lines have described the effect of cytokines on epithelial barrier functions, permeability, proliferation and apoptosis. For instance, proinflammatory TNFα decreases the expression of tight junction proteins and, subsequently, increases epithelial permeability, whilst the anti-inflammatory IL-6 decreases the permeability [44]. Apart from the physical barrier function of the intestinal epithelial cells, their important role is also in the induction of mucosal adaptive immunity which includes cooperation of epithelial cells with antigen presentation cells and lymphocytes resulting in antibody production and cellular immunity [45].

Despite our inability to determine if the changes in immunological parameters are caused by possible reduction of gestation length or unnatural hormonal changes, both caused by cloprostenol induction of farrowing, or most likely by concurrence of these effects, from a practical point of view, for the breeder, the most important fact is that these changes occur. The complexity of the net of mucosal immune responses is very sensitive to changes in cytokine levels and all interventions; in this case, induction of parturition should be applied in compelling circumstances, with adequate knowledge of the side effects and after thorough pros and cons analysis.

## Conclusion

This study confirmed the impact of farrowing induction using cloprostenol on immune parameters, namely the increased proportion of lymphocytes, lower concentrations of lysozyme and of some cytokines.

## Materials and methods

### Animals

In the experiment, 37 multiparous sows (line Topigs 40) of various parity from Czech farms were used. Nineteen sows (mean parity 3.5, range of parity 2–8) were given 175 μg of cloprostenol, an analogue of prostaglandin F2α (PGF Veyx® forte, Veyx-Pharma GmbH, Germany) intramuscularly on day 114 of gestation (induced sows). Eighteen sows (mean of parity 3.1, range of parity 2–8) were not treated (non-induced sows). All parturitions during which the samples of colostrum were taken occurred up to 24 h after cloprostenol administration. Colostrum samples from non-induced sows were taken only from sows which were farrowing on day 114 or 115 of gestation to maintain the similar length of gestation as induced sows.

### Sample collection and cell isolation

Samples of colostrum (30 mL) were collected by manual milking at the time interval between the birth of the first and fourth piglet. Colostrum was collected as a mixed sample from different teats. First two front pairs of teats were excluded from sampling to allow suckling of piglets born so far. Also, the first rear pair of teats was excluded to avoid contamination of the sample by dirt from sow’s rear legs. The teats were cleaned with 30% ethanol (Sigma) before milking. Samples were stored at 4 °C no longer than 2 h till processed.

Samples were diluted 1:2 in PBS (Lonza) and centrifuged at 600 x *g*. Supernatants were stored at − 80 °C for cytokine, lysozyme and complement assays. Pellets were resuspended in PBS and centrifuged once more at 600 x *g*. Cells were counted (Coulter Counter M4, Coulter Cientifica) and transferred into U-bottomed 96-well microtiter plates (BD Biosciences) for flow cytometry immunostaining.

### Immunostaining

Details of mAbs and second-step reagents used in this study are summarized in Table 2. In each staining step the reagents were incubated for 20 min at 4 °C. Staining of cell surface markers was performed in two steps: incubation with primary antibodies was followed by a second incubation with isotype-specific secondary antibodies. For the staining of lymphocyte subpopulations, two additional incubation steps for cell surface marker labelling were performed: free binding sites of the goat anti-mouse antibody were blocked by incubation with mouse serum (10 μl per sample). Thereafter, samples were incubated with Zenon labelled antibodies specific for CD3 (see Table 2 for details on antibodies and Zenon). Cell surface marker labelling was performed in a washing and staining buffer (WSB: PBS containing 0.2% gelatine from cold water fish skin, 1 g NaN_3_ and 1.84 g of Na_4_EDTA per litre, all reagents from Sigma–Aldrich). Following each incubation step, the cells were washed twice with 200 μl WSB. Prior to incubation and following every washing step, the cells were resuspended using a plate shaker. Moreover, single-stain samples for each fluorochrome present in various panels were prepared as compensation controls. Antigens with the highest expected expression density were selected as compensation controls. These were the following: CD3-PE, CD8-Alexa647, CD4-DyLight405, CD3-PE-TXRD and CD3-Alexa647.

**Table 2:** Primary and secondary reagents used for flow cytometry analysis

*Antigen*	*Clone*	*Isotype*	*Flourochrome*	*Labeling strategy*	*Source of primary antibody*
*Leukocyte staining panel*					
CD45	K252 1E4	IgG1	Alexa Fluor 647	Secondary antibody^a^	A generous gift from Dr. K. Haverson, University of Bristol, UK
CD172α	74–22-15	IgG2b	Alexa Fluor 488	Secondary antibody^b^	WSU Monoclonal Antibody Center
swc8	MIL3	IgM	PE	Secondary antibody^c^	A generous gift from Dr. J.K. Lunney, Animal Parasitology Institute, Beltsville, USA
*Lymphocyte subpopulations staining panel*					
CD2	PG168A	IgG3	Alexa Fluor 488	Secondary antibody^d^	WSU Monoclonal Antibody Center
CD3	PPT3	IgG1	PE	Zenon labeling^e^	BD Biosciences
CD4	74–12-4	IgG2b	DyLight405	Secondary antibody^f^	WSU Monoclonal Antibody Center
CD8α	76–2-11	IgG2a	Alexa Fluor 647	Secondary antibody^g^	WSU Monoclonal Antibody Center
γδTCR	PGBL22A	IgG1	PE-TXRD	Secondary antibody^h^	WSU Monoclonal Antibody Center

^a^Goat anti-Mouse IgG1-Alexa647, Life Technologies

^b^Goat anti-Mouse IgG2b-Alexa488, Life Technologies

^c^Goat anti-Mouse IgM- R-Phycoerythrin, Life Technologies

^d^Goat anti-Mouse IgG3-Alexa488, Life Technologies

^e^IgG1-R-Phycoerythrin Zenon labeling kit, Life Technologies

^f^Goat anti-Mouse IgG2b-DyLight405, GeneTex

^g^Goat anti-Mouse IgG2a-Alexa647, Life Technologies

^h^Goat anti-Mouse IgG1-PE-TXRD, Life Technologies

### FCM analysis

**Fig. 1:**
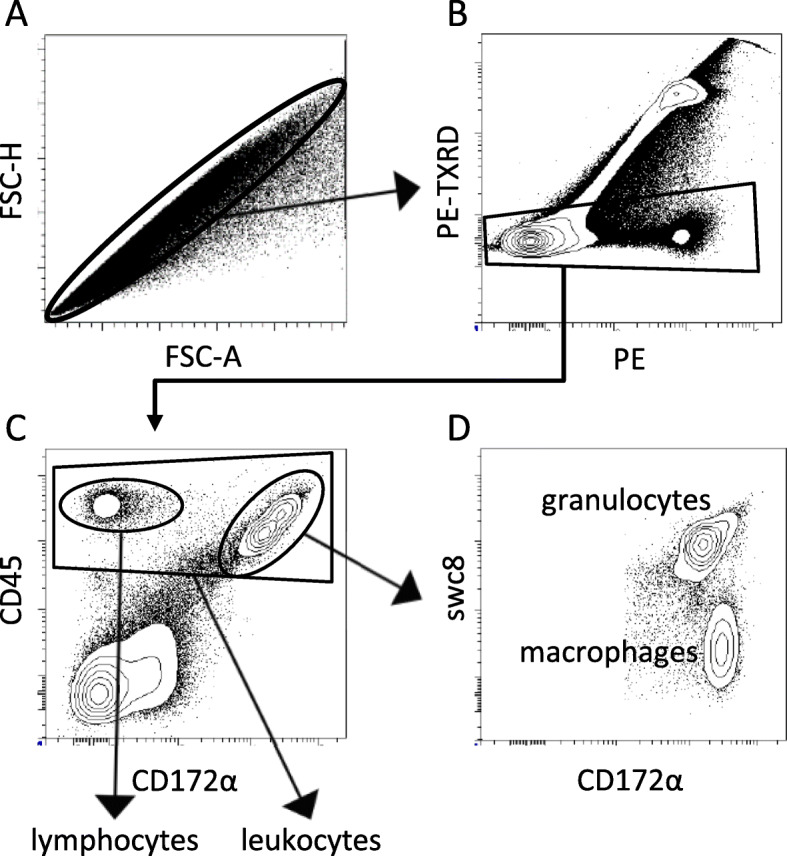
Gating strategy – analysis of leukocyte populations. Doublet events were discriminated on the basis of forward scatter integral versus forward scatter peak height (**a**). Dead cells were excluded from analysis using propidium iodide staining (**b**). Leucocytes were determined as CD45^+^ cells (**c**). Granulocytes were determined as CD45^+^CD172α^+^swc8^+^, macrophages as CD45^+^CD172α^+^swc8^−^ and lymphocytes as CD45^+^CD172α^−^swc8^−^ (**d**)

**Fig. 2:**
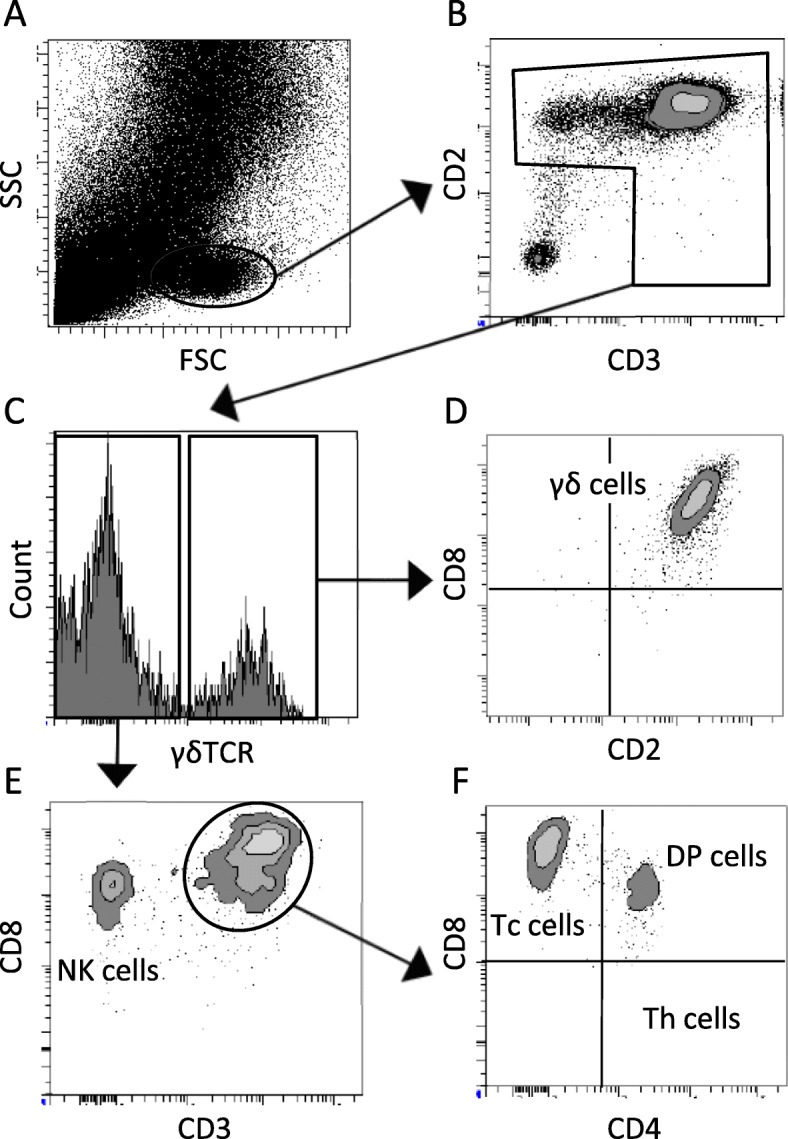
Gating strategy – analysis of lymphocyte subpopulations. Light scatter characteristics were used for rough determination of the lymphocyte gate and exclusion of debris (**a**). Lymphocytes were determined as all CD3^+^ and/or CD2^+^ cells (**b**). From all lymphocytes, the percentage of γδTCR^+^ cells (γδ T cells) was determined (**c**). γδ T cells were subdivided into three subclasses based on CD2 and CD8 expression: CD2^+^CD8^+^, CD2^+^CD8^−^, CD2^−^CD8^−^ (**d**). Individual subpopulations of γδTCR^−^ T cells were defined as follows: Th cells: CD2^+^CD3^+^CD4^+^CD8^−^γδTCR^−^; DP cells: CD2^+^CD3^+^CD4^+^CD8^+^γδTCR^−^ and Tc cells: CD2^+^CD3^+^CD4^−^CD8^hi^γδTCR^−^ (**f**). In addition to T cells, we also defined NK cells as CD2^+^CD3^−^CD4^−^CD8^lo^γδTCR^−^ (**e**)

Data were acquired using a flow cytometer (BD FACS Aria Fusion; BD Biosciences, USA) and analysed with FACSDiva 6.0 software (BD Biosciences, USA). In each measurement 500,000 events were collected and a percentage of positive cells was analysed. Doublet events were discriminated on the basis of forward scatter integral versus forward scatter peak height (Fig. 1a). Dead cells were excluded from analysis using propidium iodide staining (Fig. 1b). Leucocytes were determined as CD45^+^ cells (Fig. 1c). Granulocytes were determined as CD45^+^CD172α^+^swc8^+^ (Fig. 1d), macrophages as CD45^+^CD172α^+^swc8^−^ (Fig. 1d) and lymphocytes as CD45^+^CD172α^−^swc8^−^. Lymphocyte subpopulations were determined using a different staining panel. In this panel, light scatter characteristics were used for rough determination of the lymphocyte gate and exclusion of debris (Fig. 2a). Whole population (100%) of lymphocytes was determined as all CD3^+^ and/or CD2^+^ cells (Fig. 2b). Afterwards, from the all lymphocytes, the percentage of γδTCR^+^ cells was determined (Fig. 2c). According to Sinkora et al. [46], γδTCR^+^ cells were subdivided into three subclasses based on CD2 and CD8 expression: CD2^+^CD8^+^, CD2^+^CD8^−^, CD2^−^CD8^−^ (Fig. 2d). Individual subpopulations of γδTCR^−^ cells were defined as follows: Th cells: CD2^+^CD3^+^CD4^+^CD8^−^γδTCR^−^; DP cells: CD2^+^CD3^+^CD4^+^CD8^+^γδTCR^−^ and Tc cells: CD2^+^CD3^+^CD4^−^CD8^hi^γδTCR^−^ (Fig. 2f). In addition to T cells, we also defined NK cells as CD2^+^CD3^−^CD4^−^CD8^lo^γδTCR^−^ (Fig. 2e).

### Lysozyme concentration determination by radial diffusion

The amount of lysozyme was assessed in vitro by radial diffusion in agarose gel mixed with *Micrococcus luteus* (CCM 169). Samples of 20 μl of acellular colostrum supernatant from individual sows were applied in duplicates into the wells cut in the agarose placed in glass plates and incubated at room temperature (20 °C). After 24 h, diameter of diffusion zone was measured, and the amount of lysozyme in the sample was converted to milligram per milliliter of colostrum according to the calibration curve (for details see Poisot et al. [47]).

### Complement activity determination

The total bacteriolytic activity was determined using a modified bioluminescence-based method [48–50]. Transformed *E. coli* K12 with luxABCDE gene, originating from soil bacterium *Photorhabdus*, expressing bacterial luciferase (Lux) [51] was used. This Gram-negative bacterium is very sensitive to complement but not to lysozyme as checked before [52]. Bacterial luciferase catalyzes the oxidation of a long-chain aldehyde and the reduced flavin mononucleotide (FMNH_2_) with emission maxima at 490 nm. Bacteria were exposed to acellular colostrum supernatant (diluted 1:2 in PBS) from individual sows. As a negative control, a randomly selected sample of acellular colostrum inactivated at 65 °C for 10 min was used. Plasmid of bacteria contains genes for enzyme luciferase and its substrate — long-chain aldehyde. The light emission of the reaction is positively correlated with the viability of *E. coli* which was measured using an LM01-T luminometer (Immunotech, Czech Republic). The time (in hours) required for 50% viability of *E. coli* was evaluated (in triplicates) using kinetic curves corresponding to complement activity of each sample. There is a reciprocal proportion between time of *E. coli* viability and complement activity; the shorter time represents higher complement activity in colostrum of identical concentration. For better comprehension, the complement activity was expressed as inverted values (in h^− 1^).

### Elisa

Cytokine concentration was determined by the following ELISA kits: TGF beta 2 Pig ELISA kit, TNF alpha Pig ELISA Kit, Interleukin 6 Pig ELISA Kit, Interleukin 1β Pig ELISA Kit, Interleukin 4 Pig ELISA Kit. Interleukin 10 Pig ELISA Kit (all Abcam) and Granulocyte-Macrophage colony-stimulating factor (CSF2) ELISA Kit (CUSABIO). All assays were performed according to the manufacturers’ protocols. All the samples were run in duplicates.

### Data analysis

Data are expressed as the mean ± SD. Statistical analysis was performed with the Mann-Whitney U-test (comparison of cell proportions, lysozyme and complement activity in samples from induced and non-induced sows) and Independent two-sample t-test (comparison of cytokine concentrations in samples from induced and non-induced sows) using data analysis software (Statistica, StatSoft, Inc.). The obtained data were considered significant if *P*-values were *P* < 0.05.

## Data Availability

The datasets used during the current study are available from the corresponding author on reasonable request.
